# Efficacy and Safety of Robotic Surgery vs. Open Surgery for Hilar Cholangiocarcinoma: A Comprehensive Review

**DOI:** 10.7759/cureus.66790

**Published:** 2024-08-13

**Authors:** Sparsh Dixit, Chanrashekhar Mahakalkar, Shivani Kshirsagar, Akansha Hatewar

**Affiliations:** 1 Surgery, Jawaharlal Nehru Medical College, Datta Meghe Institute of Higher Education and Research, Wardha, IND; 2 Surgery, Jawaharlal Nehru Medical College, Datta Meghe Institute of Higher Education and Research, wardha, IND

**Keywords:** postoperative recovery, oncological outcomes, surgical resection, open surgery, robotic surgery, hilar cholangiocarcinoma

## Abstract

Hilar cholangiocarcinoma, a rare and aggressive bile duct malignancy, presents significant challenges in surgical management. Traditionally treated with open surgery, the emergence of robotic surgery has introduced a new dimension to surgical approaches for this condition. This review aims to systematically compare the efficacy and safety of robotic surgery versus open surgery for hilar cholangiocarcinoma. We conducted a comprehensive review of the literature, including clinical studies, case series, and comparative analyses of robotic and open surgical techniques. Data on oncological outcomes, functional recovery, survival rates, complications, and cost-effectiveness were extracted and analyzed to provide a detailed comparison of the two surgical approaches. Robotic surgery offers several potential advantages over open surgery, including reduced intraoperative blood loss, smaller incisions, and shorter recovery times. However, it requires specialized training and has a higher initial cost. Open surgery, while more established and broadly practiced, remains associated with longer recovery periods and higher complication rates. Oncological outcomes, such as R0 resection rates and survival, appear comparable between the two approaches, though robotic surgery may offer improvements in functional recovery and postoperative quality of life. Both robotic and open surgery have their merits in the treatment of hilar cholangiocarcinoma. Robotic surgery presents promising benefits in terms of reduced invasiveness and improved recovery, while open surgery continues to be a reliable and well-established option. The choice of surgical approach should be guided by patient-specific factors, surgeon expertise, and institutional resources. Further research is needed to refine surgical techniques and establish long-term outcomes, which will aid in optimizing treatment strategies for this challenging malignancy.

## Introduction and background

Hilar cholangiocarcinoma, also known as Klatskin tumor, is a rare but aggressive malignancy that arises from the epithelial cells of the bile ducts at the confluence of the right and left hepatic ducts [1]. It constitutes approximately 50%-70% of all cholangiocarcinomas and is distinguished by its location at the hepatic hilum, where the bile ducts exit the liver. The global incidence of cholangiocarcinoma has been increasing, with notable geographical variations due to differences in risk factors and diagnostic capabilities [2]. The disease is more prevalent in Southeast Asia, largely due to liver fluke infections, while in Western countries, it is often associated with primary sclerosing cholangitis, biliary stones, and other hepatobiliary conditions [3]. The pathogenesis of hilar cholangiocarcinoma involves a complex interplay of genetic mutations, chronic inflammation, and environmental factors. The tumor typically exhibits a desmoplastic reaction, characterized by dense fibrous stroma, which can result in biliary obstruction and various complications [4]. Clinically, patients usually present with symptoms such as jaundice, pruritus, abdominal pain, and weight loss. The insidious onset of these symptoms often leads to a delayed diagnosis, and many patients present with advanced disease at the time of diagnosis, complicating treatment and management [5].

Surgical resection remains the cornerstone of curative treatment for hilar cholangiocarcinoma. The complex anatomy of the hepatic hilum, along with the frequent involvement of critical vascular structures, makes surgical intervention particularly challenging [6]. However, achieving complete resection with negative margins (R0 resection) is vital for long-term survival and is the only potentially curative option for this malignancy. The prognosis of patients significantly improves when the tumor is entirely removed without leaving any residual disease [6]. Open surgery has been the traditional approach for the resection of hilar cholangiocarcinoma. It involves a laparotomy, allowing the surgeon to directly visualize and palpate the tumor and the surrounding structures. The procedure often includes major liver resections and biliary reconstructions, which require significant surgical expertise and experience [7]. In recent years, robotic surgery has emerged as a minimally invasive alternative. Utilizing robotic systems, surgeons can perform complex procedures with enhanced precision and dexterity. The robotic approach offers several potential benefits over open surgery, including reduced blood loss, smaller incisions, and shorter recovery times. However, it requires specialized training and has a steep learning curve, limiting its widespread adoption [8].

This comprehensive review aims to systematically compare the efficacy and safety of robotic surgery versus open surgery for treating hilar cholangiocarcinoma. The review will evaluate key outcomes such as oncological efficacy, functional recovery, survival rates, complication profiles, and cost-effectiveness. By synthesizing the current evidence, the review seeks to thoroughly assess both surgical approaches. The comparison of robotic and open surgery is crucial for clinical decision-making, as it will help clinicians determine the most appropriate treatment strategy for their patients. Given the increasing adoption of robotic surgery and the inherent challenges in treating hilar cholangiocarcinoma, a critical evaluation of the benefits and risks associated with each surgical method is essential. This review will also identify gaps in current knowledge and highlight areas where further research is needed, ultimately contributing to advancing care for patients with this challenging and complex malignancy.

## Review

Surgical techniques and approaches

Open surgery has traditionally been the standard treatment for hilar cholangiocarcinoma, a complex condition due to the intricate anatomy of the biliary tree and surrounding structures. Surgical techniques for this condition have significantly advanced over time, with improvements in instruments and methods enhancing patient outcomes [9]. The standard procedure typically involves a right subcostal incision, often extended with a midline incision, to gain sufficient access to the hepatic hilum and biliary structures. This surgery generally includes resection of the extrahepatic bile duct and gallbladder, lymphadenectomy of the hepatoduodenal ligament, and, in many cases, an extended hemihepatectomy to ensure clear margins and optimal oncological control [10]. Postoperative management following open surgery is crucial for recovery and involves vigilant monitoring for potential complications. Common concerns include bile leaks, bleeding, and liver failure, which can significantly impact recovery [11]. Patients may require biliary drainage and nutritional support during their hospital stay, and recovery often demands extended hospital stays and rehabilitation. Despite these challenges, open surgery remains a well-established option, especially in cases necessitating extensive resection [11].

**Figure 1:**
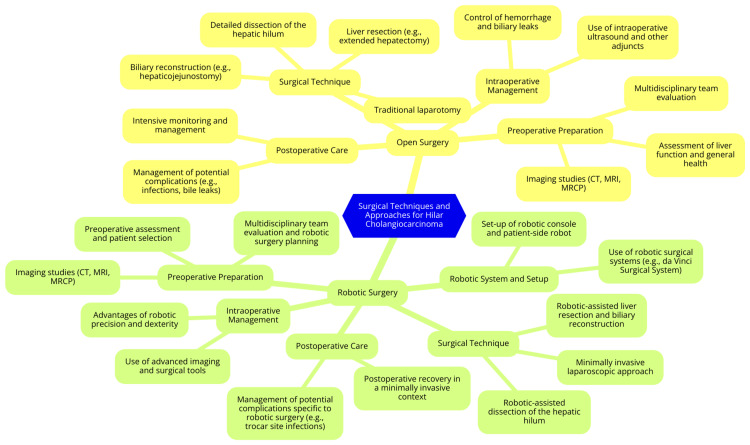
Surgical techniques and approaches Image Credit: Dr. Sparsh Dixit

In recent years, robotic surgery has emerged as a promising minimally invasive alternative to open surgery for hilar cholangiocarcinoma. Technological advancements in robotic systems have provided surgeons with enhanced 3D visualization and magnification and improved dexterity and precision for complex dissection and reconstruction [12]. These features make robotic surgery particularly advantageous for procedures involving the delicate anatomy of the biliary tree. The robotic approach generally follows similar surgical steps to those of open surgery, including resection of the extrahepatic bile duct, lymphadenectomy, and, if necessary, liver resection. However, robotic systems allow for smaller incisions, potentially reducing postoperative pain and faster recovery [13]. Despite these benefits, the successful implementation of robotic surgery requires specialized training and a steep learning curve for surgeons. Mastering the robotic platform and addressing the unique challenges of hilar cholangiocarcinoma is essential for achieving optimal outcomes. Studies have shown that robotic surgery can result in acceptable operative times, controlled blood loss, and favorable postoperative outcomes [12]. Nevertheless, further research is needed to directly compare robotic surgery's long-term oncological results with traditional open surgery. As the field progresses, robotic techniques may increasingly play a significant role in the surgical management of hilar cholangiocarcinoma, offering patients a viable alternative with the potential for improved recovery and outcomes [12]. Surgical techniques and approaches are shown in Figure 1.

Efficacy comparison

The comparison of efficacy between robotic and open surgery for hilar cholangiocarcinoma involves several key domains, including oncological outcomes, functional outcomes, survival outcomes, and long-term outcomes [14]. Regarding oncological outcomes, robotic surgery has demonstrated R0 resection rates comparable to open surgery. Studies on robotic-assisted radical resection report R0 resection rates of approximately 74.3%, reflecting effective tumor removal with negative margins similar to traditional methods [15]. Additionally, robotic surgery generally examines more lymph nodes than open surgery, which is crucial for accurate staging and assessing disease spread. Adequate lymph node dissection is associated with improved survival outcomes. Recurrence rates are similar between robotic and open surgery, with studies showing three-year disease-free survival rates of about 39.5% for robotic surgery and 35.3% for open surgery, indicating that oncological control is maintained with both techniques [16]. Regarding functional outcomes, robotic surgery may offer advantages in preserving liver function due to its minimally invasive nature, which typically results in less liver trauma compared to open surgery. This is particularly relevant for patients with pre-existing liver conditions or those undergoing extensive liver resections [17]. Additionally, postoperative quality-of-life assessments often show that robotic surgery patients experience faster recovery and greater comfort. The minimally invasive approach generally reduces postoperative pain and a quicker return to normal activities, enhancing overall patient satisfaction [18]. When evaluating survival outcomes, overall survival rates for patients undergoing robotic surgery are comparable to those undergoing open surgery. Studies indicate no significant difference in overall survival rates, with figures around 76.7% for robotic surgery versus 72.9% for open surgery at the three-year mark [19]. Similarly, disease-free survival rates are not significantly different between the two approaches, suggesting that while robotic surgery may improve recovery and reduce complications, it does not compromise long-term oncological outcomes [20]. Regarding long-term outcomes, the impact of robotic surgery on liver regeneration is still under investigation. However, the minimally invasive nature of robotic procedures is believed to facilitate better liver regeneration compared to open surgery, which can be more traumatic. Monitoring and follow-up protocols remain crucial for both surgical approaches. Patients undergoing robotic surgery may benefit from shorter follow-up intervals due to quicker recovery and return to baseline function. However, regular oncological assessments are still necessary to monitor for recurrence [21].

Safety and complications

Intraoperative factors are crucial for evaluating the safety and efficacy of robotic versus open surgery for hilar cholangiocarcinoma. One significant consideration is blood loss and transfusion rates. Robotic surgery is generally associated with lower blood loss compared to open surgery, which can reduce the need for blood transfusions [22]. This advantage is significant in complex procedures where maintaining hemodynamic stability is critical. However, robotic surgeries often have longer operative times, frequently exceeding 600 minutes, whereas open surgeries may be completed more quickly. This extended operative time is due to the complexity of robotic techniques and the need for meticulous dissection in the challenging anatomy of hilar cholangiocarcinoma [23]. Technical difficulties and troubleshooting are also important aspects of robotic procedures. While robotic systems offer enhanced precision and visualization, they can present unique challenges, such as equipment malfunctions or the need for intraoperative adjustments. These technical issues can complicate the procedure and may be less prevalent in traditional open surgeries, where surgeons rely on tactile feedback and direct visualization [8]. Postoperative complications are a critical factor in assessing surgical safety. Mortality rates following robotic surgery for hilar cholangiocarcinoma are generally low, around 1.8%, which is comparable to or better than some outcomes associated with open surgery [12]. However, morbidity rates can vary. Robotic surgery has shown a morbidity rate of approximately 39.8%, which may include complications such as infections, bile leaks, and vascular issues. These rates indicate that while robotic surgery can be effective, it has risks, and careful monitoring is essential [12]. The length of hospital stays and recovery time are another important factor. Patients undergoing robotic surgery typically experience shorter hospital stays than those with open surgery. This shorter recovery period is largely due to the minimally invasive nature of robotic techniques, which often results in less postoperative pain and quicker functional recovery [24]. Each surgical approach has its own set of risks. Open surgery is associated with larger incisions, which can lead to increased postoperative pain, longer recovery times, and higher rates of complications such as wound infections. The extensive nature of open surgery can also contribute to longer hospital stays, which may increase the risk of hospital-acquired infections [25]. On the other hand, robotic surgery introduces risks related to the technology itself. Potential equipment failures and complications may arise from the learning curve associated with mastering robotic systems. Additionally, inadvertent intraoperative hypothermia has been noted as a concern in robotic surgeries, potentially leading to further complications if not properly managed [26].

Cost-effectiveness and resource utilization

The cost comparison between robotic surgery and traditional open surgery reveals notable differences, particularly regarding initial investments and procedure expenses. The acquisition of robotic surgical systems, such as the da Vinci system, typically exceeds $1.5 million, with annual service contracts often surpassing $100,000. This significant initial investment requires a high volume of procedures to justify the costs. To achieve a break-even point, a robotic system would need to be used for over 300 procedures annually over seven years, resulting in an average cost of more than $1,000 per patient [27]. Regarding cost per procedure, robotic surgeries are generally more expensive than laparoscopic and open surgeries. For example, data from gallbladder surgeries show that the average total cost for robotic procedures is about $21,756, compared to $17,779 for open surgery and approximately $15,520 for laparoscopic approaches [28]. The higher costs associated with robotic surgery are primarily due to the high price of robotic instruments, which can exceed $1,500 per instrument and have a limited number of uses-typically around 10 per instrument. Additionally, ongoing maintenance costs, including annual service contracts, contribute to the overall financial burden of robotic surgery [29]. Robotic surgery generally requires more operating room time compared to traditional methods. Studies indicate that robotic surgeries can range from 54.6 to 328.7 minutes, whereas laparoscopic procedures typically last between 50.2 and 260 minutes. This extended operative time can increase costs and resource allocation within the surgical setting [29]. Furthermore, implementing robotic surgery requires dedicated staff training and specialized equipment, which can strain hospital resources. Despite these challenges, robotic and laparoscopic approaches often lead to shorter hospital stays than open surgery, potentially offsetting some initial costs through reduced inpatient care requirements [8]. While the upfront costs of robotic surgery are significantly higher, potential long-term economic benefits may enhance its cost-effectiveness. Improved patient outcomes, such as fewer postoperative complications, can decrease long-term healthcare costs [30]. Studies have shown that robotic colorectal and urological procedures are associated with fewer complications than their open and laparoscopic counterparts. However, further research is needed to comprehensively assess the long-term cost-effectiveness of robotic surgery, considering factors such as patient recovery, quality of life, and overall healthcare utilization [31].

Patient and surgeon perspectives

Patient satisfaction is a critical factor in evaluating surgical interventions, especially in the context of robotic surgery for hilar cholangiocarcinoma. Many patients report enhanced postoperative pain management and overall comfort with robotic procedures compared to traditional open surgeries. The minimally invasive nature of robotic techniques usually results in smaller incisions, which can lead to reduced postoperative pain and quicker recovery times. While anecdotal evidence suggests these benefits, more comprehensive studies are needed to directly compare pain scores and analgesic requirements between robotic and open surgical approaches for hilar cholangiocarcinoma [32]. Patient-reported outcomes and experiences are also vital indicators of surgical success. Although specific research on patient experiences following robotic surgery for hilar cholangiocarcinoma is limited, existing literature on minimally invasive surgery indicates that patients often express higher satisfaction levels due to shorter hospital stays and faster returns to normal activities. However, the impact of robotic surgery on quality of life and functional outcomes for this patient population remains an area for further exploration. Understanding these perspectives can help refine surgical practices and improve patient care [33]. The successful implementation of robotic surgery for hilar cholangiocarcinoma depends significantly on the surgeon’s experience and expertise. Training for robotic surgery is extensive, requiring surgeons to be proficient in traditional hepatobiliary surgical techniques and the specific nuances of robotic systems. The learning curve for robotic hepatectomy is estimated to involve approximately 20 to 30 cases, although this may vary significantly for complex procedures such as those involving hilar cholangiocarcinoma. Ensuring that surgeons receive adequate training and proctoring is crucial for maintaining high standards of safety and efficacy in these challenging surgeries [34]. Surgeon preference also significantly influences the adoption of robotic techniques. Factors such as prior experience with open and minimally invasive surgeries, access to robotic platforms, and institutional support can greatly affect a surgeon's decision to offer robotic surgery. When robotic systems are readily available, surgeons may be more inclined to perform minimally invasive procedures, potentially leading to a shift in practice patterns. Ultimately, the interplay between patient satisfaction, surgeon experience, and the evolving landscape of surgical technology will shape the future of robotic surgery for hilar cholangiocarcinoma and other complex surgical conditions [35].

Current guidelines and recommendations

Current international and national guidelines provide a framework for robotic surgery in treating hilar cholangiocarcinoma, though they do not explicitly recommend it over traditional open surgery [36]. The Society of American Gastrointestinal and Endoscopic Surgeons (SAGES) has published guidelines emphasizing proper training and credentialing of surgeons performing robotic procedures. However, these guidelines do not specifically address robotic surgery for hilar cholangiocarcinoma [37]. Similarly, the British Association of Urological Surgeons (BAUS) has developed a robotic surgery curriculum that outlines a five-stage training pathway. Although this curriculum primarily focuses on urological procedures, it highlights the importance of standardized training for robotic surgery across various surgical specialties [38]. The American Urological Association (AUA) has also issued standard operating procedures for urologic robotic surgery, detailing minimum requirements for granting robotic privileges, including proctoring and monitoring outcomes. However, these guidelines do not extend to hepatobiliary surgeries. While the role of robotic surgery is increasingly recognized in various fields, specific guidelines for its use in hilar cholangiocarcinoma still need to be expanded [39]. Institutional protocols and practices regarding robotic surgery for hilar cholangiocarcinoma vary significantly across different healthcare settings. A consensus document from the SAGES-MIRA Robotic Surgery Consensus Group highlights the need for standard criteria for training nurses, technicians, and surgeons in robotic surgery, reflecting the diversity in institutional practices. Some institutions have begun implementing structured training curricula incorporating simulators and proficiency-based progression (PBP) methodologies to standardize outcomes in robotic surgery. However, adopting such comprehensive training programs is inconsistent, and many institutions still need more formalized protocols [12]. Moreover, the success of robotic surgery programs often depends on institutional support, which includes dedicated operating rooms, specialized nursing teams, coordinators, and maintenance resources. The availability and allocation of these resources can vary significantly between healthcare settings, impacting the overall effectiveness and safety of robotic procedures. As robotic surgery advances, understanding these institutional variations is crucial for optimizing patient outcomes and establishing best practices in treating hilar cholangiocarcinoma [40].

Future directions and research gaps

The future of robotic surgery is set for transformative changes driven by technological advancements, the necessity for rigorous comparative research, and a shift toward personalized surgical approaches. Significant technological innovations have already enhanced the precision and capabilities of robotic surgery. Developments such as highly dexterous robotic arms, miniaturized instruments, and advanced imaging technologies have improved surgical navigation and accuracy. Additionally, integrating artificial intelligence (AI) and machine learning is revolutionizing surgical decision-making by enabling better recognition of complex anatomical structures and facilitating autonomous functions during surgery [8]. Robotic surgery is expected to evolve towards smaller, more intelligent systems with greater autonomy. The potential emergence of microrobots capable of interacting with pathologies at the cellular level could revolutionize minimally invasive techniques. These advancements are anticipated to improve patient outcomes, including faster recovery times and reduced complication rates. Enhanced dexterity and precision of robotic systems allow for more delicate surgical maneuvers, minimizing tissue damage and improving overall surgical efficacy [8]. Despite these promising advancements, there is a critical need for more robust comparative effectiveness research, particularly randomized controlled trials (RCTs) that directly compare robotic surgery with traditional open surgery. Such studies are essential to establish clear evidence of the benefits and limitations of robotic techniques, especially in terms of long-term outcomes and cost-effectiveness. Long-term follow-up studies are necessary to evaluate the durability of surgical outcomes achieved through robotic surgery. Current data often focus on short-term results; comprehensive long-term studies will provide insights into recurrence rates, survival outcomes, and quality of life post-surgery, which are crucial for assessing the true efficacy of robotic interventions [41]. The personalization of surgical approaches is gaining traction in robotic surgery, emphasizing developing patient selection criteria that optimize surgical outcomes. Identifying which patients are best suited for robotic procedures can enhance efficacy and minimize risks, tailoring interventions to individual patient profiles based on tumor characteristics, anatomical considerations, and overall health status. Integrating robotic surgery with personalized medicine represents a significant frontier in surgical innovation. This approach involves using genetic, biomarker, and other patient-specific data to inform surgical decisions, potentially leading to more effective and tailored interventions. Combining advanced robotic techniques with personalized treatment plans could significantly enhance surgical precision and improve patient outcomes [42].

## Conclusions

In conclusion, this comprehensive review provides a thorough comparison of robotic and open surgery for hilar cholangiocarcinoma, emphasizing the relative efficacy, safety, and cost-effectiveness of each approach. While robotic surgery offers advantages such as reduced blood loss, smaller incisions, and quicker recovery times, it also presents challenges related to its steep learning curve and higher costs. On the other hand, open surgery remains a well-established method with proven outcomes, particularly in achieving R0 resection. However, it is associated with greater postoperative discomfort and longer recovery periods. The choice between these surgical techniques should be tailored to individual patient factors, including tumor characteristics, patient comorbidities, and the surgeon's expertise. This review underscores the importance of a nuanced and patient-centered approach in the surgical management of hilar cholangiocarcinoma, highlighting the need for further research to refine these techniques and improve patient outcomes.
